# Supplementary material for: Assessment of Berries of Some Sea Buckthorn Genotypes by Physicochemical Properties and Fatty Acid Content of the Seed
Source: Plants (Basel). 2022 Dec 7;11(24):3412. doi: 10.3390/plants11243412 (PMC9782847; doi:10.3390/plants11243412)
Supplement: Supplementary file 1 [file plants-11-03412-s001.zip › plants-2041425-supplementary.pdf]

plants

MDPI

Article

Supplementary Materials: Assessment of Berries of some Sea Buckthorn Genotypes by Physicochemical Properties and Fatty Acid Content of the Seed

Mónika Máté[1], Granit Selimaj[1,2], Gergely Simon[2], Lilla Szalóki-Dorkó[1], Gitta Ficzek[2*]

[1]Department of Fruit and Vegetable Processing Technology, Institute of Food Science and Technology, Hungarian University of Agriculture and Life Sciences

[2]Department of Fruit Growing, Institute of Horticulture, Hungarian University of Agriculture and Life Sciences

* Correspondence: ficzek.gitta@uni-mate.hu

Table S1. Detailed list of analytes, compound names, trivial names, retention times (Rt) [min], and resolution (R) [-]

Abbr.	Compound	Trivial Name	R_t [min]	R [-]
C14:0	Methyl myristate	Myristic acid	9.94	39.0
C15:0	Methyl pentadecanoate	Pentadecylic acid	11.05	14.4
C16:0	Methyl palmitate	Palmitic acid	12.43	15.8
C16:1n-7c	Methyl palmitoleate	Palmitoleic acid	13.24	21.8
C18:0	Methyl stearate	Strearic acid	16.30	24.8
C18:1n-9c	Methyl oleate	Oleic acid	17.20	6.0
C18:2n-6c	Methyl linoleate	Linoleic acid	19.04	2.6
C18:3n-3c	Methyl linoleate	α-linolenic acid	21.56	17.6
C20:0	Methyl arachidate	Arachidic acid	21.95	5.9
C20:1n-9c	Methyl eicosanoate	Gondoic acid	23.00	15.0
C20:3n-6c	Methyl eicosatrienoate	dihomo-γ-linolenic acid	26.63	18.6
C22:0	Methyl behenate	Behenic acid	27.91	10.2
C24:1n-9c	Methyl nervonate	Nervonic acid	31.02	8.2

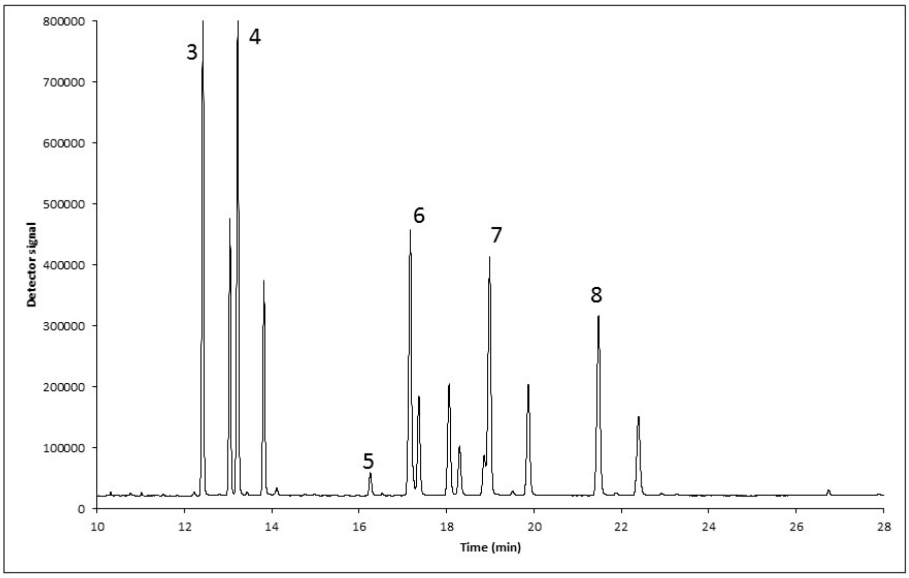

Figure S1. Chromatograms of R-01 candidate. (Peak 3: Palmitic acid; Peak 4: Palmitoleic acid; Peak 5: Strearic acid; Peak 6: Oleic acid; Peak 7: Linoleic acid; Peak 8: α-linolenic acid)
